# Effectiveness of the use of an oscillating positive expiratory pressure device in bronchiectasis with frequent exacerbations: a single-arm pilot study

**DOI:** 10.3389/fmed.2023.1159227

**Published:** 2023-05-12

**Authors:** So Rae Kim, Sun-Hyung Kim, Geun-Hyeong Kim, Jun Yeun Cho, Hayoung Choi, Hyun Lee, Seung Won Ra, Ki Man Lee, Kang Hyeon Choe, Yeon-Mok Oh, Yoon Mi Shin, Bumhee Yang

**Affiliations:** ^1^Department of Internal Medicine, Chungbuk National University Hospital, Chungbuk National University College of Medicine, Cheongju, Republic of Korea; ^2^Department of Biochemistry, Chungbuk National University College of Medicine, Cheongju, Republic of Korea; ^3^Division of Pulmonary and Critical Care Medicine, Department of Internal Medicine, Chungbuk National University Hospital, Chungbuk National University College of Medicine, Cheongju, Republic of Korea; ^4^Artificial Intelligence Center, Chungbuk National University Hospital, Cheongju, Republic of Korea; ^5^Division of Pulmonary, Allergy, and Critical Care Medicine, Department of Internal Medicine, Hallym University Kangnam Sacred Heart Hospital, Seoul, Republic of Korea; ^6^Division of Pulmonary Medicine and Allergy, Department of Internal Medicine, Hanyang University College of Medicine, Seoul, Republic of Korea; ^7^Division of Pulmonary Medicine, Department of Internal Medicine, Ulsan University Hospital, University of Ulsan College of Medicine, Ulsan, Republic of Korea; ^8^Department of Pulmonary and Critical Care Medicine, Asan Medical Center, University of Ulsan College of Medicine, Seoul, Republic of Korea

**Keywords:** bronchiectasis, oscillating positive expiratory pressure, acute exacerbation, sputum volume, physiotherapy

## Abstract

Impaired airway clearance in patients with non-cystic fibrosis bronchiectasis causes frequent bacterial infection, chronic inflammation, and progressive tissue destruction. We aimed to evaluate whether an oscillating positive expiratory pressure (OPEP) device could allow effective sputum expectoration and prevent acute exacerbations in patients with bronchiectasis who had frequent acute exacerbations. This open-label, single-arm, prospective study included 17 patients who experienced three or more acute exacerbations in the past year. We evaluated the prevention of acute exacerbations, subjective symptom improvement, and change in sputum amount during the use of the Aerobika (Trudell Medical International, London, ON) OPEP device twice daily for 6  months. Of all enrolled patients, only two acute exacerbations occurred during the study period, indicating a significant decrease compared with the number of acute exacerbations before the device use (*p* < 0.001). Additionally, Bronchiectasis Health Questionnaire score changed from 58.7 to 66.6, showing significant improvement over the treatment period (*p* < 0.001). The largest sputum volume was observed 3  months after OPEP device use (baseline: 10 ml, 3rd month 25 ml, *p* = 0.325). There were no major adverse events related to the use of OPEP devices. Twice-daily physiotherapy with OPEP device in patients with bronchiectasis who have frequent exacerbations may facilitate symptomatic improvement and prevention of acute exacerbations without serious adverse events.

## Introduction

Non-cystic fibrosis bronchiectasis (hereafter referred to as bronchiectasis) is a chronic respiratory disease characterized by irreversible bronchial dilatation and chronic respiratory symptoms such as cough, sputum expectoration, and dyspnea (1). Dysfunction of airway clearance disrupts normal host defense, and leads to frequent infection, chronic inflammation of the bronchial tree, and progressive remodeling and destruction of the airways. The emerging concept of vortex model suggests that each component of pathophysiological process interacts with all the other components, which might better explain the heterogeneity of non-cystic fibrosis bronchiectasis and its response to various treatments (2, 3). Accordingly, the treatment goal of bronchiectasis seeks to reduce respiratory symptoms and prevent exacerbations by promoting airway clearance (1).

There are several airway clearance techniques (ACT) for effective sputum expectoration, including the active cycle of breathing technique, autogenic drainage, postural drainage, positive expiratory pressure (PEP) devices use, high-frequency chest wall oscillation, and intrapulmonary percussion ventilation (4). Oscillating positive expiratory pressure (OPEP) devices are among the most frequently prescribed ACTs in patients with bronchiectasis in United States (5). They can improve airway clearance in patients with bronchiectasis, and therefore reduce subjective respiratory symptoms; improve the quality of life; and reduce acute exacerbations (AEs) in stable bronchiectasis patients (6–10). However, there are limited data on the role of OPEP devices in the prevention of AEs in patients with history of frequent exacerbations, which is an important therapeutic goal for bronchiectasis.

Therefore, we aimed to evaluate the preventive effect of an OPEP device on AEs in patients with bronchiectasis who had frequent AEs. Further, we investigated whether the OPEP device could improve subjective respiratory symptoms in patients with bronchiectasis.

## Materials and methods

### Participants

We included patients with bronchiectasis at Chungbuk National University Hospital registered in the Korean Multicenter Bronchiectasis Audit and Research Collaboration (KMBARC) registry. The KMBARC is a prospective, non-interventional, observational cohort study of bronchiectasis in Korea (11, 12). Patients were enrolled from August 2021 to January 2022. We included patients who experienced three or more AEs within 1 year. AE of bronchiectasis was defined as worsening of three or more major symptoms lasting ≥ 48 h, which resulted in a change in treatment (13). The main symptoms included coughing, change in sputum volume/viscosity, sputum suppuration, dyspnea, exercise ability, fatigue, malaise, and hemoptysis. Patients were doing prior drainage techniques such as active cycle of breathing and autogenic drainage as trained by their physician. Patients were instructed to continue prior method as well as oscillating PEP devices. The exclusion criteria were as follows: (1) prior use of PEP or other PEP devices, (2) history of pneumothorax, and (3) history of hemoptysis and active bleeding in the previous year. The study protocol was reviewed and approved by the Institutional Review Board of Chungbuk National University Hospital (IRB no 2021-09-013-001). This study was conducted in accordance with the amended Declaration of Helsinki. Written informed consent was obtained from all patients at the time of enrollment.

### Study design

**Figure 1:**
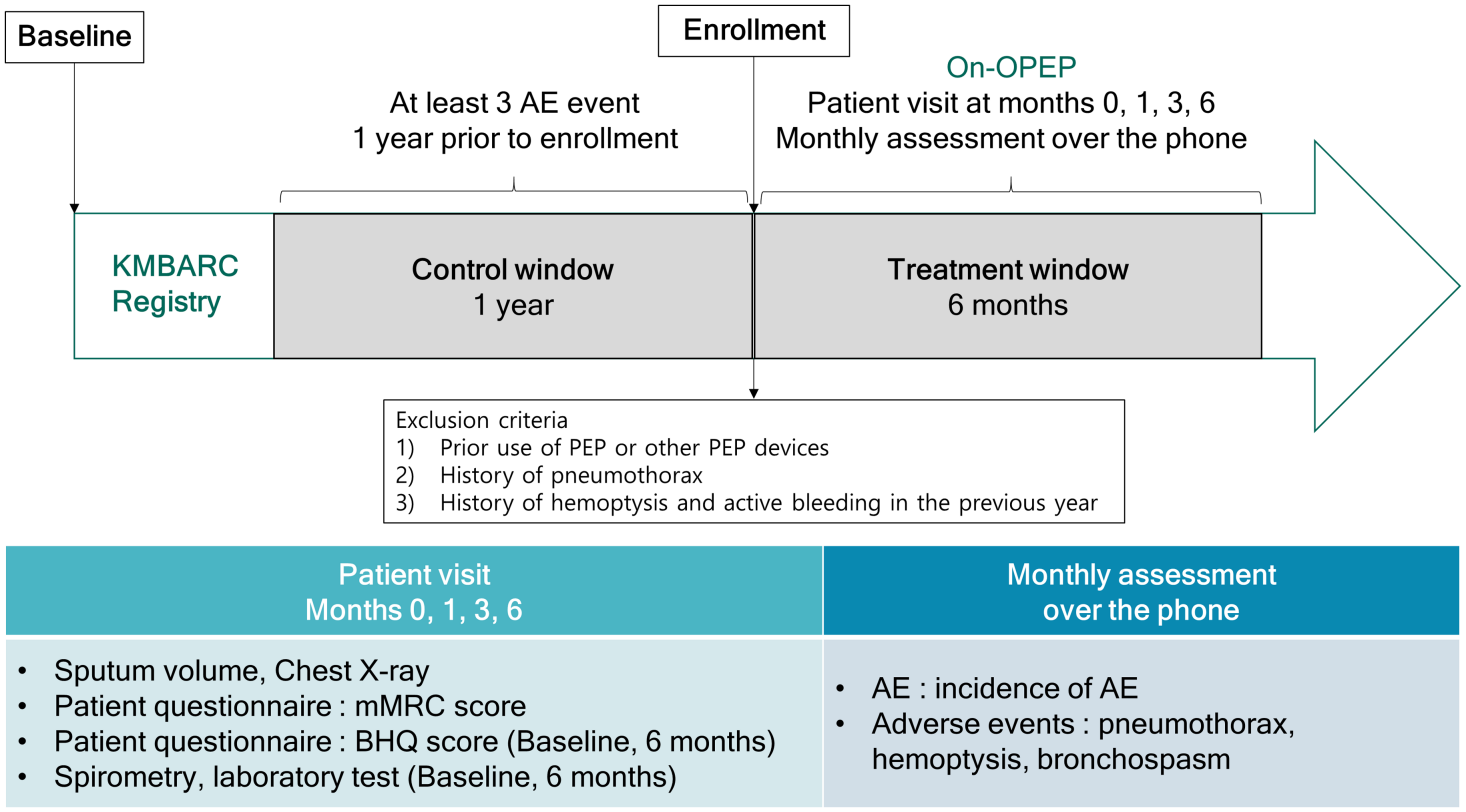
Study design. AE, acute exacerbation; OPEP, oscillating positive expiratory pressure device; KMBARC, Korean Multicenter Bronchiectasis Audit and Research Collaboration; mMRC, modified Medical Research Council; BHQ, Bronchiectasis Health Questionnaire.

This open-label, single-arm, prospective study evaluated the efficacy of an OPEP device. Participants were instructed to use the Aerobika device (Trudell Medical International, London, ON) twice a day for 6 months (Figure 1). Each session was defined as 10–20 blows into the device with 2–3 huffs at the end of the session. Patients were instructed to repeat the session for 10–20 minutes, and the entire procedure was performed twice a day. Resistance setting for the devices were varied from 1–5 as prescribed by the physician. Participants made study visits at baseline as well as at 1, 3, and 6 months after enrollment. At each visit, we measured the dyspnea score (modified Medical Research Council [mMRC]) and sputum volume. At baseline and the last visit, results of the patient questionnaire (Bronchiectasis Health Questionnaire [BHQ] score), pulmonary function and blood tests were obtained. Additionally, patients received monthly phone calls for obtaining information regarding AEs and adverse events.

### Measurements

Baseline demographic characteristics included age, sex, body mass index (weight in kilograms divided by height in meters squared), and smoking status (never, former, or current smoker). Symptoms were evaluated using the mMRC and validated Korean version of the BHQ for quality of life (14–16). The radiological severity of bronchiectasis was measured using the modified Reiff score (17). Chest computed tomography was used to evaluate the radiological extent of bronchiectasis. The FACED (forced expiratory volume in 1 s [FEV_1_] % predicted, age, chronic colonization, extension, and dyspnea) and Bronchiectasis Severity Index scores were calculated to assess the clinical status and severity of bronchiectasis as previously described (18, 19).

The specimens underwent microbiological analyses according to standard methods (20). Conventional semi-qualitative bacterial and fungal cultures were obtained. All samples underwent initial Gram staining before sputum culture if they met the Murray and Washington criteria (21).

The pulmonary function test was performed at the time of patient enrollment and 6 months after the enrollment. Pre-bronchodilator and post-bronchodilator spirometry were performed following the American Thoracic Society/European Respiratory Society criteria (22). The absolute values of FEV_1_ and forced vital capacity (FVC) were recorded; additionally, the percentages of predicted values for FEV_1_ and FVC were calculated with an automatic calculator using a reference equation obtained from a representative Korean sample (23). Normal ventilation was defined as pre-bronchodilator FEV_1_/FVC ≥ 0.70 and FVC ≥ 80% predicted. Obstructive ventilatory disorder was defined as a pre-bronchodilator FEV_1_/FVC < 0.70. Restrictive ventilatory disorder was defined as FEV_1_/FVC ≥ 0.7 and FVC < 80% predicted.

### Oscillating positive expiratory pressure device

This study used Aerobika (Trudell Medical International, London, ON) as the OPEP. Aerobika is a drug-free handheld mechanical OPEP device that can help clear excess mucus in the airways and improve breathing. Moreover, Aerobika helps in airway opening by creating positive airway pressure when the patient exhales (24). It induces vibrations in the airways that thin and loosen mucus, allowing the mucus to naturally move to the upper airways where it may be easily coughed out (25). Patients were evaluated for exhalation resistance setting and educated on how to use Aerobika by trained nurse during each visit.

### Outcomes

Primary outcome was cumulative number of acute exacerbation events during the 6 months study period. We also evaluated subjective symptoms improvement measured by BHQ score and sputum amount change as a secondary outcomes.

### Adverse events

Oscillating PEP devices are not recommended in patients with neuromuscular weakness, recent head and neck surgery or trauma, active hemoptysis, untreated pneumothorax, and middle ear disease; we checked chest X-ray for pneumothorax on every patient visit and made monthly phone calls to check for the occurrence of any possible adverse events as mentioned above. Patients were asked if they were diagnosed with or treated for pneumothorax or hemoptysis during the observational period.

### Statistical analysis

Continuous variables were expressed as medians and interquartile ranges, while categorical variables were expressed as numbers (%). Continuous variables were compared using the Mann–Whitney *U*-test while categorical variables were compared using Pearson’s chi-squared test or Fisher’s exact test. The Wilcoxon matched-paired rank test was used to compare the effects of OPEP device use. All tests were two-sided. Statistical significance was set at *p* < 0.05. All statistical analyses were performed using the IBM SPSS Statistics for Windows (version 27.0; IBM Corp., Armonk, NY, United States).

## Results

### Patient characteristics

Table 1 shows the characteristics of the 17 patients with frequent AEs of bronchiectasis. The median age of the 17 patients was 62.0 years (interquartile range [IQR], 52.0–71.0 years), 8 patients (47.1%) were male, and 12 patients (70.6%) were never-smokers. The median body mass index was 21.8 (IQR, 21.0–23.8). The most common comorbidity was chronic obstructive pulmonary disease (COPD)/asthma (41.2%), followed by cardiovascular disease (23.5%). Three patients (17.6%) had history of tuberculosis. Microorganisms were identified in 52.9% of the patients, with *Pseudomonas aeruginosa* (35.3%) being the most common. The median modified Reiff, FACED, and Bronchiectasis Severity Index scores were 6 (IQR, 3–10), 2 (IQR, 1–2), and 9 (IQR, 7–10), respectively. Additionally, the median baseline BHQ score was 59 (IQR, 51–65). Before device use, patients had experienced exacerbations at least 3 times (IQR, 3–3) over the previous last year. The overall follow-up period was 182 days.

**Table 1:** Baseline characteristics.

	*N* = 17
Age, years	62 (57–71)
Sex, male	8 (47.1)
BMI, kg/m^2^	21.8 (21.0–23.8)
Smoking history	
Never-smoker	12 (70.6)
Current- or ex-smoker	5 (29.4)
*Comorbidities*	
COPD/Asthma	7 (41.2)
Cardiovascular disease	4 (23.5)
History of tuberculosis	3 (17.6)
Diabetes Mellitus	1 (5.9)
Chronic liver disease	1 (5.9)
Malignancy	1 (5.9)
Chronic kidney disease	1 (5.9)
**Microbiology**	**9 (52.9)**
*Pseudomonas aeruginosa*	6 (35.3)
*Haemophilus influenzae*	1 (5.9)
*Klebsiella pneumoniae*	1 (5.9)
*Staphylococcus aureus*	1 (5.9)
**Spirometry**	
FVC, L	2.4 (1.9–2.8)
FVC, % predicted	74 (66–78)
FEV1, L	1.8 (1.2–2.1)
FEV1, % predicted	70 (58–82)
FEV1/FVC ratio	72 (63–83)
**Modified Reiff score**	6 (3–10)
**FACED score**	2 (1–2)
**BSI score**	9 (7–10)
**Laboratory findings**	
WBC count/uL	7,420 (5,840–9,230)
Neutrophil count/uL	4,578 (2,808–5,258)
Eosinophil count/uL	142 (102–252)
hs-CRP, mg/dL	0.4 (0.1–1.2)
**mMRC**	1 (1–2)
**BHQ score**	58.7 (56.1–66.7)
**Sputum volume, mL**	10 (6–25)
**Previous history of AE**	3 (3–3)
**Follow up duration, days**	182 (173–193)

Data are presented as the median (interquartile range) or numbers (%).

BMI, body mass index; COPD, chronic obstructive pulmonary disease, FVC, forced vital capacity; FEV_1_, forced expiratory volume in 1 s; FACED, Forced expiratory volume in 1 s (F), age (A), presence of chronic colonization by *Pseudomonas aeruginosa* (*P. aeruginosa*, C), radiological extension (number of pulmonary lobes affected) (E), dyspnea (D); *BSI, Bronchiectasis* Severity Index; WBC, White blood cell; hs-CRP, high-sensitivity C-reactive protein; mMRC, modified Medical Research Council; BHQ score, The Bronchiectasis Health Questionnaire; AE, acute exacerbation.

### Outcomes

**Figure 2:**
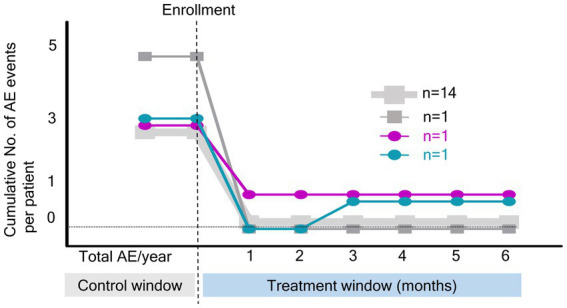
Cumulative number of acute exacerbation events during the study period. NO, number; AE, acute exacerbation.

**Figure 3:**
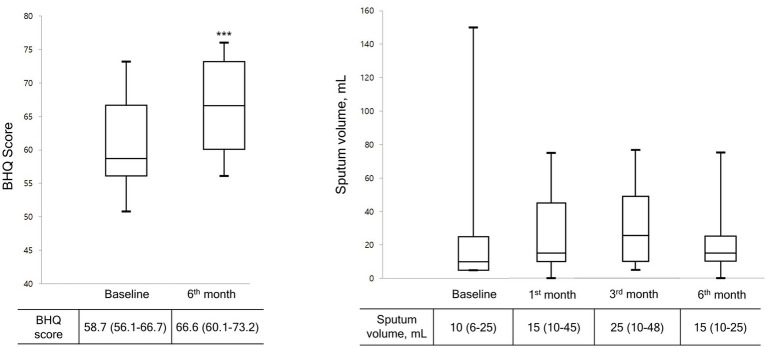
Changes in BHQ score and sputum volume during the study period. Data are presented as the median (interquartile range). Wilcoxon matched-paired rank test was used to compare pre and post treatment. BHQ score, The Bronchiectasis Health Questionnaire Higher scores indicate better quality of life.

Figure 2 shows the cumulative number of AEs in patients. Only two AEs occurred during the study period, indicating a significant decrease compared with the number of AEs before the OPEP device use (*p* < 0.001). As shown in Figure 3, the BHQ score changed from 58.7 (IQR, 56.1–66.7) to 66.6 (IQR, 60.1–73.2) showing significant improvement over the intervention period (*p* < 0.001). The sputum volume was largest at 3 months after OPEP device use (median 25 ml, IQR, 10–48). There was no significant post-intervention change in the laboratory test and spirometry results except FEV1, L (See Supplementary file 1).

### Adverse events

There were three adverse effects related to the use of an OPEP device (Table 2). All adverse events were hemoptysis. Two patients dropped out from the study due to hemoptysis (*n* = 1) and poor adherence (*n* = 1).

**Table 2:** Adverse events during study periods.

	Total	1 month (*N* = 17)	2 months (*N* = 17)	3 months (*N* = 16)	4 months (*N* = 15)	5 months (*N* = 15)	6 months (*N* = 15)
Pneumothorax	0	0	0	0	0	0	0
Hemoptysis	3	0	0	1	0	0	2
Bronchospasm	0	0	0	0	0	0	0

## Discussion

To our knowledge, this is the first study to investigate AE prevention *via* use of an OPEP device in patients with bronchiectasis. We found that the use of an OPEP device twice daily in patients with bronchiectasis who had frequent exacerbations significantly prevented further occurrence of AEs during OPEP device use without any severe adverse events. Additionally, it allowed subjective symptom improvement, as indicated by the BHQ score.

Several studies have investigated the utility of OPEP device in respiratory diseases (25, 26). OPEP device use can prevent AEs and allow various beneficial effects in patients with COPD. A study on 50 patients with stable COPD reported that the use of an OPEP device for 2 years reduced AEs compared with the control group (27). Another study performed on 120 patients with stable COPD reported improved dyspnea scores, subjective symptoms, and lung function parameters as well as reduced exacerbation after 6 months of treatment with an OPEP device (28). Furthermore, real-world data has shown that adjunctive therapy with an OPEP device can effectively prevent AE in patients with COPD who have a history of exacerbation (29). However, there are limited data regarding the prevention of AE in patients with bronchiectasis. The present study shows that the use of an OPEP device can help prevent AE in patients with bronchiectasis who frequently experience exacerbations.

A systematic review of seven studies involving 146 patients with bronchiectasis revealed that the use of an OPEP device facilitated sputum expectoration and improved the quality of life compared with no treatment; however, it did not reduce the exacerbation rate (30). In patients with stable bronchiectasis or AEs of bronchiectasis, compared with other ACTs, therapy with an OPEP device showed similar effects on sputum expectoration, lung function, and subjective symptoms. However, the OPEP group showed higher patient acceptability and preference (31–33). Unlike previous studies, this study evaluated the efficacy of an OPEP device in patients with the frequent exacerbator phenotype, but not in stable patients or patients with current AEs. Frequent exacerbations are the strongest predictor of future exacerbations (34). Moreover, since AEs can decrease the quality of life and increase mortality, it is important to reduce further exacerbations in patients with the frequent exacerbation phenotype.

The beneficial effect of AE prevention in our study could be attributed to effective sputum expectoration. Similar effects have been reported in patients with sputum-producing COPD. Daily use of an OPEP device has been shown to facilitate sputum expectoration in patients with sputum-producing COPD and improve symptoms. Moreover, effective ventilation improves pulmonary function and exercise capacity (24, 35). OPEP device also has been shown to increase the sputum volume in patients with bronchiectasis (36–38). However, its effects on AE were not evaluated. We observed a gradual increase in the sputum volume, which peaked 3 months after OPEP use. The reduction of AEs could be attributed to the increased sputum volume and decreased inflammation (1).

Two patients stopped using the device before the study protocol ended. One patient stopped using the device because the patient did not feel the need to use the device anymore due to lack of sputum after using the OPEP device. The other patient withdrew from the study had blood-tinged sputum and while the device was temporarily withheld, patient was hospitalized for severe acute respiratory syndrome coronavirus 2 (SARS-CoV-2) infection and stopped using the device ever since.

This study has several limitations. First, this was a relatively small-scale study and was not a controlled clinical trial with a control group in which physiotherapy with an OPEP device was omitted. Prior studies regarding the effects of oscillating devices were also conducted in small sample. (39, 40) This study is a pilot study, and we are planning to further conduct a clinical trial based on this pilot study. Second, patients referred to the tertiary hospital due to frequent AEs may have been affected by various prior treatments. Third, the participants’ adherence rate could only be determined from self-reporting; however, the patients’ use of the OPEP device was examined at every visit. Moreover, the patients’ subjective symptom improvement may have contributed to good adherence. Finally, we did not observe changes in spirometry such as improvement in FEV1, which have been previously reported. However, 6 months is relatively short to evaluate disease progression or functional change; therefore, further studies are warranted to evaluate the long-term effect of an OPEP device use.

## Conclusion

In conclusion, physiotherapy with an OPEP device in patients with bronchiectasis who have frequent exacerbations may facilitate subjective symptom improvement and AE prevention without severe adverse events. However, further large-scale studies are warranted on the effectiveness of physiotherapy with an OPEP device.

## Author’s note

Plain language summary: Twice-daily physiotherapy with an oscillating positive expiratory pressure (OPEP) airway clearance device in patients with bronchiectasis who have frequent exacerbations may facilitate symptomatic improvement and prevention of recurrent episodes of exacerbations without serious adverse events.

## Data availability statement

The raw data supporting the conclusions of this article will be made available by the authors, without undue reservation.

## Ethics statement

The studies involving human participants were reviewed and approved by Institutional Review Board of Chungbuk National University Hospital. The patients/participants provided their written informed consent to participate in this study.

## Author contributions

SK and S-HK: design and methodology, data collection, data analysis, and writing. G-HK: expertise, data analysis and feedback. JC, KL, and KC: design and methodology, expertise and feedback. HC, HL, SR, and Y-MO: expertise, writing, and feedback. YS: expertise, feedback, acquisition of funding, and writing. BY: design and methodology, acquisition of funding, data collection, data analysis, and writing. All authors contributed to the article and approved the submitted version.

## Funding

This work was supported by the research grant of the Chungbuk National University in 2021 and supported by grants from the National Research Foundation of Korea (2020R1A5A2017476). Also, this work was supported by the research grant of the Chungbuk National University Hospital in 2021.

## Conflict of interest

The authors declare that the research was conducted in the absence of any commercial or financial relationships that could be construed as a potential conflict of interest.

## Publisher’s note

All claims expressed in this article are solely those of the authors and do not necessarily represent those of their affiliated organizations, or those of the publisher, the editors and the reviewers. Any product that may be evaluated in this article, or claim that may be made by its manufacturer, is not guaranteed or endorsed by the publisher.
