# Supplementary material for: Effectiveness of the use of an oscillating positive expiratory pressure device in bronchiectasis with frequent exacerbations: a single-arm pilot study
Source: Front Med (Lausanne). 2023 May 12;10:1159227. doi: 10.3389/fmed.2023.1159227 (PMC10213442; doi:10.3389/fmed.2023.1159227)
Supplement: Supplementary file 1 [file Table_1.docx]

**Additional file 1.** Comparison of spirometry and laboratory findings at the initial and 6^th^ month study

|  | Initial | 6^th^ month | P-value |
| --- | --- | --- | --- |
| **Spirometry** |  |  |  |
| FVC, L | 2.4 (1.9-2.8) | 2.2 (1.9-2.7) | 0.266 |
| FVC, % predicted | 74 (66-78) | 71 (67-81) | 0.863 |
| FEV1, L | 1.8 (1.2-2.1) | 1.6 (1.3-2.0) | 0.021 |
| FEV1, % predicted | 70 (58-82) | 65 (52-80) | 0.141 |
| FEV1/FVC ratio | 72 (63-83) | 71 (61-78) | 0.110 |
| **Laboratory findings** | N=15 | N=10 |  |
| WBC count, /uL | 7420 (5840-9230) | 7255 (6223-8625) | 0.078 |
| Neutrophil count, /uL | 4578 (2808-5258) | 4144 (3443-5685) | 0.641 |
| Eosinophil count, /uL | 142 (102-252) | 140 (70-310) | 0.461 |
| hs-CRP, mg/dL | 0.4 (0.1-1.2) | 0.4 (0.2-0.7) | 0.742 |

Data are presented as the median (interquartile range) or numbers (%).

Wilcoxon matched-paired rank test was used to compare pre and post treatment.

FVC, forced vital capacity; FEV_1_, forced expiratory volume in 1 second; WBC, White blood cell; hs-CRP, high-sensitivity C-reactive protein.
